# Flash monitor initiation is associated with improvements in HbA_1c_ levels and DKA rates among people with type 1 diabetes in Scotland: a retrospective nationwide observational study

**DOI:** 10.1007/s00125-021-05578-1

**Published:** 2021-10-07

**Authors:** Anita Jeyam, Fraser W. Gibb, John A. McKnight, Joseph E. O’Reilly, Thomas M. Caparrotta, Andreas Höhn, Stuart J. McGurnaghan, Luke A. K. Blackbourn, Sara Hatam, Brian Kennon, Rory J. McCrimmon, Graham Leese, Sam Philip, Naveed Sattar, Paul M. McKeigue, Helen M. Colhoun, on behalf of the Scottish Diabetes Research Network Epidemiology Group

**Affiliations:** 1 MRC Institute of Genetics and Cancer (formally known as Institute of Genetic and Molecular Medicine), University of Edinburgh, Edinburgh, UK; 2 Edinburgh Centre for Endocrinology & Diabetes, Royal Infirmary of Edinburgh, Edinburgh, UK; 3 Western General Hospital, NHS Lothian, Edinburgh, UK; 4 Queen Elizabeth University Hospital, Glasgow, UK; 5 Division of Molecular and Clinical Medicine, University of Dundee, Dundee, UK; 6 Ninewells Hospital, Dundee, UK; 7 Grampian Diabetes Research Unit, Diabetes Centre, Aberdeen Royal Infirmary, Aberdeen, UK; 8 Institute of Cardiovascular and Medical Sciences, University of Glasgow, Glasgow, UK; 9 Usher Institute of Population Health Sciences and Informatics, Centre for Population Health Sciences, School of Molecular, Genetic and Population Health Sciences, University of Edinburgh, Edinburgh, UK; 10 Public Health, NHS Fife, Kirkcaldy, UK

**Keywords:** Diabetes mellitus type 1, Flash monitoring, HbA_1c_, Hypoglycaemia, Ketoacidosis

## Abstract

**Aims/hypothesis:**

We assessed the real-world effect of flash monitor (FM) usage on HbA_1c_ levels and diabetic ketoacidosis (DKA) and severe hospitalised hypoglycaemia (SHH) rates among people with type 1 diabetes in Scotland and across sociodemographic strata within this population.

**Methods:**

This study was retrospective, observational and registry based. Using the national diabetes registry, 14,682 individuals using an FM at any point between 2014 and mid-2020 were identified. Within-person change from baseline in HbA_1c_ following FM initiation was modelled using linear mixed models accounting for within-person pre-exposure trajectory. DKA and SHH events were captured through linkage to hospital admission and mortality data. The difference in DKA and SHH rates between FM-exposed and -unexposed person-time was assessed among users, using generalised linear mixed models with a Poisson likelihood. In a sensitivity analysis, we tested whether changes in these outcomes were seen in an age-, sex- and baseline HbA_1c_-matched sample of non-users over the same time period.

**Results:**

Prevalence of ever-FM use was 45.9% by mid-2020, with large variations by age and socioeconomic status: 64.3% among children aged <13 years vs 32.7% among those aged *≥*65 years; and 54.4% vs 36.2% in the least-deprived vs most-deprived quintile. Overall, the median (IQR) within-person change in HbA_1c_ in the year following FM initiation was −2.5 (−9.0, 2.5) mmol/mol (−0.2 [−0.8, 0.2]%). The change varied widely by pre-usage HbA_1c_: −15.5 (−31.0, −4.0) mmol/mol (−1.4 [−2.8, −0.4]%) in those with HbA_1c_ > 84 mmol/mol [9.8%] and 1.0 (−2.0, 5.5) mmol/mol (0.1 [−0.2, 0.5]%) in those with HbA_1c_ < 54 mmol/mol (7.1%); the corresponding estimated fold change (95% CI) was 0.77 (0.76, 0.78) and 1.08 (1.07, 1.09). Significant reductions in HbA_1c_ were found in all age bands, sexes and socioeconomic strata, and regardless of prior/current pump use, completion of a diabetes education programme or early FM adoption. Variation between the strata of these factors beyond that driven by differing HbA_1c_ at baseline was slight. No change in HbA_1c_ in matched non-users was observed in the same time period (median [IQR] within-person change = 0.5 [−5.0, 5.5] mmol/mol [0.0 (−0.5, 0.5)%]). DKA rates decreased after FM initiation overall and in all strata apart from the adolescents. Estimated overall reduction in DKA event rates (rate ratio) was 0.59 [95% credible interval (CrI) 0.53, 0.64]) after FM vs before FM initiation, accounting for pre-exposure trend. Finally, among those at higher risk for SHH, estimated reduction in event rates was rate ratio 0.25 (95%CrI 0.20, 0.32) after FM vs before FM initiation.

**Conclusions/interpretation:**

FM initiation is associated with clinically important reductions in HbA_1c_ and striking reduction in DKA rate. Increasing uptake among the socioeconomically disadvantaged offers considerable potential for tightening the current socioeconomic disparities in glycaemia-related outcomes.

**Graphical abstract:**

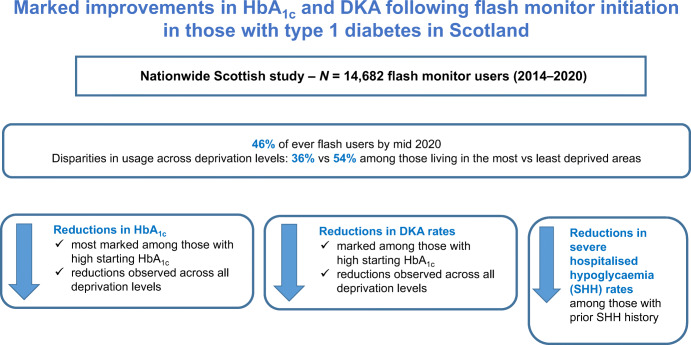

**Supplementary Information:**

The online version contains peer-reviewed but unedited supplementary material available at 10.1007/s00125-021-05578-1.

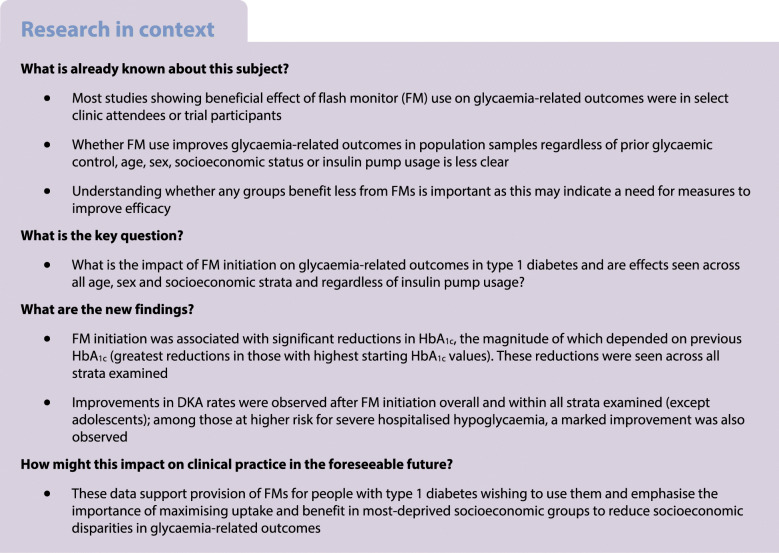



## Introduction

In type 1 diabetes, there has been a shift from traditional methods of self-monitoring of blood glucose using fingerpricks and glucometers (compliance can be poor with <50% adherence to guidelines among people with type 1 diabetes in Sweden [1]) to using new technologies that allow for more frequent measurements with less discomfort. These new technologies enable real-time or intermittently scanned continuous glucose monitoring [2]. The latter is known as flash monitoring, with the only system currently available for use in the National Health Service (NHS) in the UK (including Scotland) being Abbott’s Freestyle Libre. Flash monitors (FMs) have been available in the UK since late 2014 [3]. They became freely available in Scotland from the NHS in 2018, having been only self-funded previously. Eligibility for FM use follows a mixture of criteria defined by each of the Scottish Health boards.

The largest RCT of FMs (*N* = 328), IMPACT, demonstrated a significant effect of FM use on hypoglycaemia without any significant change in HbA_1c_. However IMPACT was restricted to adults with good glycaemic control (HbA_1c_ ≤ 58 mmol/mol [7.5%]) [4], and is therefore not representative of the range of current recipients of this technology from the NHS. Observational studies have shown reductions in HbA_1c_, diabetic ketoacidosis (DKA) and hypoglycaemia with use of FMs [5–14]. Greater effects on HbA_1c_ have been found in individuals with high initial HbA_1c_ but, apart from this, study of variation in effectiveness across different subgroups of recipients has been limited, particularly for DKA, where there is a gap in the literature. It is important to determine whether any groups benefit less from FMs, as this may indicate a need for measures to improve efficacy.

In this paper, we aimed to describe the contemporary prevalence of FM use among all those with type 1 diabetes in Scotland and to examine the association of FM initiation with glycaemic outcomes (HbA_1c_, DKA and hypoglycaemia) across the full range of recipients and within age, sex and socioeconomic groups, as well as by prior glycaemic control, insulin pump usage and completed diabetes education programme. We also examined outcomes among the early adopters, who self-funded the device before it became NHS-funded.

## Methods

### Data sources

We used anonymised data from the Scottish Care Information - Diabetes Collaboration (SCI-DC) database, a registry with extensive electronic health records for all those with diabetes in Scotland. These routinely collected data include start and end dates for FM use, as well as prescription data. These data are also linked to hospital admissions data SMR01 from Information Services Division Scotland and mortality data from National Records of Scotland (NRS). The database and linkage procedure have been described in detail elsewhere [15, 16].

### Study population

Among all those alive with type 1 diabetes, observable at any point between 2014 and mid-2020, we included for glycaemic outcome analyses those who started using an FM between 2014 and 31 October 2019 to limit the number of recipients with no post-initiation HbA_1c_ by the end of study date. The type of diabetes was ascertained based on a validated algorithm [15]. FM start and stop dates were assessed from SCI-DC device dates and from encashed prescription data for Libre sensors. Individuals contributed person-time from the latest of either date of diabetes diagnosis or start of observability in the Scottish diabetes registry, to the earliest of date of death, last date of observability, first stop-date of FM use or 30 June 2020 (end of study). Glycaemic measures were assessed up to a maximum of 5 years prior to FM initiation, hence individual person-time was left-censored 5 years before FM initiation date. To disentangle the effect of FM initiation from that of other devices, person-time was right-censored at the first start date of insulin pump/continuous glucose monitoring (CGM) device if these started after FM initiation.

### Exposure, outcomes and covariates

The exposure of interest was FM usage. Individual person-time was partitioned into intervals of 1 year centred on the date of FM initiation [17]. HbA_1c_ records were obtained from the SCI-DC data. Individuals’ median HbA_1c_ over time slices was used for analyses. Baseline value was defined as median over the 2 years prior to FM initiation for continuous covariates, and most severe state over this time window for discrete covariates.

Baseline HbA_1c_ was categorised to reflect different levels of glycaemic control (in mmol/mol [%]: <54 [7.1]; ≥54 [7.1] to ≤63 [7.9]; ≥64 [8.0] to ≤74 [8.9]; ≥75 [9.0] to ≤84 [9.8]; >84 [9.8]). Data on hospitalisations and deaths for DKA and severe hypoglycaemia from up to 5 years pre-FM initiation were obtained using the ICD-10 codes (http://apps.who.int/classifications/icd10/browse/2016/en) detailed in electronic supplementary material (ESM) Methods, anywhere on the discharge summary or cause of death.

Area-level deprivation was measured by the Scottish Index of Multiple Deprivation (SIMD) 2016 definition [18], which is based on the postcode of residence. SIMD quintiles were used for analyses, Q1 being the most deprived. Insulin pump/CGM exposure and completed diabetes education programme status were ascertained from SCI-DC. Prior pump usage was defined as any usage of insulin pump preceding the initiation of FM, regardless of whether usage continued post-FM. An early adopter was defined as anyone who started FM before 2018.

### Statistical analyses

Comparison of outcomes between users and non-users of FM may be subject to allocation bias or confounding by indication. Therefore, our analyses focused on changes within users over time in outcomes from pre- to post-initiation of FM. All analyses were conducted using R version 3.6.0–64 bit [19] and at significance level 0.05. No imputation of missing data was performed.

#### HbA_1c_

Absolute within-person change from baseline HbA_1c_ was described over time, overall and among the groups of interest listed above. The significance of reductions was assessed using a one-sided (difference < 0) Wilcoxon signed-rank test with a Bonferroni correction for multiple comparisons of various time points vs baseline.

To account for any background trend over time occurring in HbA_1c_ prior to FM initiation and for repeated measurements within individuals, we used mixed models adjusted for time, age, diabetes duration at initiation, sex and baseline HbA_1c_ [20] (see ESM Methods). Specifically, log-transformed HbA_1c_ was modelled using linear mixed models, with a random intercept and time slope on the individual, with categorical FM exposure time as a covariate, implemented in *nlme 3.1-143* [21]. Model estimates represent change in HbA_1c_ compared with what the levels would have been had any pre-exposure trend continued (i.e. the counterfactual). To examine whether the association of FM with HbA_1c_ varied across groups of interest, we compared models with and without the FM × group interaction term using likelihood ratio tests (LRTs). Further, to examine whether any such interactions were explained by variation in baseline HbA_1c_ across strata, we tested whether interactions remained significant when models were adjusted for the interaction term FM × baseline HbA_1c_.

#### DKA and SHH

Crude DKA and severe hospitalised hypoglycaemia (SHH) rates were described in pre- and post-FM person-time. Due to their discrete and rare nature, DKA and SHH event rates were modelled using generalised linear mixed models with a Poisson likelihood and a random intercept on the individual, with FM exposure as a binary time-varying covariate and adjusting for pre-FM time trend. To avoid reliance on approximations of intractable integrals, these models were implemented in a Bayesian Framework using *rstan 2.19.3*, with results expressed as rate ratios with 95% credible intervals (CrIs).

Stratified analyses of DKA rates were conducted across the groups of interest. Due to the sparser nature of SHH events, we focused on high risk groups for this outcome: those with a prior history of SHH in the 5 years pre-FM and those with baseline HbA_1c_ < 54 mmol/mol (7.1%).

#### Sensitivity analyses

To ensure that any changes in outcomes attributed to FM use were not confounded by the occurrence of some more general phenomena coinciding with FM introduction, we performed crude sensitivity analyses of changes over a similar time period in a sample of non-users, matched 1:1 by sex, baseline HbA_1c_ band and age band at FM initiation date (ESM Methods). Non-users were defined as individuals who had never used a device by the user’s date of FM initiation and for at least 6 months thereafter. The significance of differences in HbA_1c_ was assessed using a one-sided Wilcoxon signed-rank test with a Bonferroni correction for multiple comparison. Comparisons of event rates were made using crude rate ratios.

## Results

The study sample-size flowchart is shown in ESM Fig. 1.

### Prevalence of FM use

The crude prevalence of ever-FM users among those alive with type 1 diabetes increased rapidly after reimbursement began, from 3.1% in 2017 to 45.9% (*n* = 14,682) by mid-2020. Usage was higher in female vs male individuals and in younger vs older age bands (Table 1). Quarterly prevalence by year and age band is detailed in ESM Fig. 2. Prevalence of use decreased with HbA_1c_ band, was higher among those with vs without prior pump usage, those with prior SHH history, and in those from least vs most-deprived areas. These disparities were present across age bands and sex, although differences were smaller in younger vs older age bands (63.7% vs 52.8% in <13 years band; 22.3% vs 43.8% in ≥65 years band).

**Table 1:** Prevalence of FM usage by mid-2020, overall and within strata of interest

Characteristic	Prevalence (%)
Overall	45.9
Age band	
<13 years	64.3
13–18 years	62.0
19–24 years	47.7
25–44 years	47.6
45–64 years	43.2
≥65 years	32.7
Sex	
Female	50.5
Male	42.2
SIMD quintile	
1	36.2
2	45.2
3	46.9
4	49.5
5	54.4
HbA_1c_ band	
<54 mmol/mol (<7.1%)	58.6
54–63 mmol/mol (7.1–7.9%)	61.8
64–74 mmol/mol (8.0–8.9%)	56.4
75–84 mmol/mol (9.0–9.8%)	49.7
>84 mmol/mol (>9.8%)	42.3
Ever insulin pump/CGM usage	
No	40.3
Yes	74.8
Ever DKA admission in past 5 years	
No	45.7
Yes	46.3
Ever SHH admission in past 5 years	
No	38.6
Yes	72.6

### Baseline characteristics of ever-users of FM

We included for analyses of glycaemic outcomes 12,256 FM users who started using the device before 31 October 2019. Their baseline characteristics are described in ESM Table 1 alongside those of matched non-users. The median FM initiation date was 16 November 2018. The median (IQR) post-FM initiation follow-up time was 1.5 (1.0, 2.0) years. Among the FM users, 23.4% had initiated a pump prior to FM, and 0.5% (*n* = 60) had stepped down from a CGM; 29.2% had a record of any completed diabetes education and 7.6% were early adopters.

### Changes in HbA_1c_

#### Overall

**Fig. 1:**
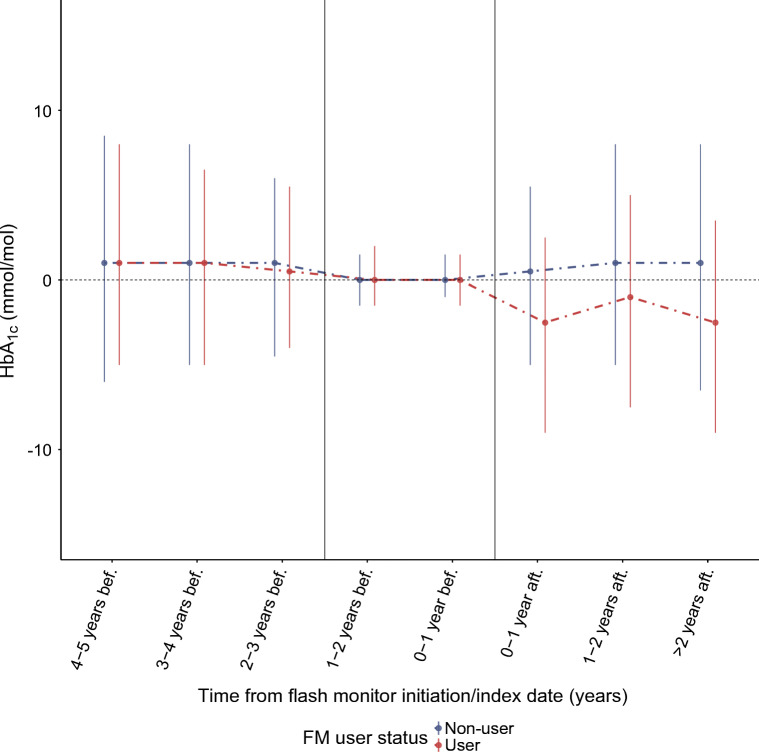
Within-person change from baseline in HbA_1c_ over time from FM initiation/index date for users vs matched non-users. Data are median (IQR). bef., before FM initiation; aft., after FM initiation. The two vertical lines denote the baseline window

Among all users combined there was a median (IQR) reduction in HbA_1c_ of −2.5 (−9.0, 2.5) mmol/mol (−0.2 [−0.8, 0.2]%) (*n* = 10,761; *p* < 0.01) within the first year post-exposure and −2.5 (−9.0, 3.5) mmol/mol (−0.2 [−0.8, 0.3]%) (*n* = 758; *p* < 0.01) for ≥2 years of exposure (Table 2). Over similar time periods, there was no change in HbA_1c_ in the matched non-users, as illustrated in Fig. 1 (median [IQR] within-person change = 0.5 [−5.0, 5.5] mmol/mol [0.0 (−0.5, 0.5)%]). Taking into consideration the slight downward trend occurring in HbA_1c_ among users prior to FM initiation, modelled estimates revealed a fold change in HbA_1c_ of 0.94 (0.94, 0.95) at 1 year post-initiation and 0.99 (0.98, 1.00) at ≥2 years (ESM Table 2).

#### Stratified analyses

Since approximately half the FM users had more than 1 year of follow-up post-exposure, results for stratified analyses focus on the year following FM exposure. Results beyond that time period are given in the tables for informative purposes. We did not perform analyses stratified by prior CGM usage due to the low number of prior CGM users.

#### By baseline HbA_1c_

Among FM users, change in HbA_1c_ was strongly dependent on HbA_1c_ at baseline, ranging from a median (IQR) reduction of −15.5 (−31.0, −4.0) mmol/mol (−1.4 [−2.8, −0.4]%) during the first year following FM initiation in those with HbA_1c_ > 84 mmol/mol (9.8%) at baseline to a slight median (IQR) increase of 1.0 (−2.0, 5.5) mmol/mol (0.1 [−0.2, 0.5]%) in those with HbA_1c_ < 54 mmol/mol (7.1%) at baseline (Table 2). Taking into consideration trends occurring in HbA_1c_ among users prior to FM initiation, the modelled estimates ranged from a fold change (95% CI) of 0.77 (0.76, 0.78) in those with HbA_1c_ > 84 mmol/mol (9.8%) at baseline to 1.08 (1.07, 1.09) in those with HbA_1c_ < 54 mmol/mol at baseline (ESM Table 2).

**Table 2:** Absolute within-person differences in HbA_1c_ with respect to baseline over time from FM initiation, overall and stratified by baseline HbA_1c_

Time from FM initiation (years)	Overall	<54 mmol/mol (7.1%)	54–63 mmol/mol (7.1–7.9%)	64–74 mmol/mol (8.0–8.9%)	75–84 mmol/mol (9.0–9.8%)	>84 mmol/mol (>9.8%)
Pre-FM						
4–5 years	1.00 (−5.00, 8.00); 9081	5.00 (0.50, 11.50); 1157	3.00 (−2.00, 8.50); 2606	0.50 (−5.00, 7.50); 2636	−1.50 (−9.00, 6.00); 1369	−6.50 (−17.50, 4.00); 1313
	0.1 (−0.5, 0.7)	0.5 (0.0, 1.1)	0.3 (−0.2, 0.8)	0.0 (−0.5, 0.7)	−0.1 (−0.8, 0.5)	−0.6 (−1.6, 0.4)
3–4 years	1.00 (−5.00, 6.50); 9520	4.00 (−0.50, 9.50); 1253	2.00 (−2.50, 7.31); 2764	0.50 (−5.00, 6.00); 2760	−1.00 (−8.00, 6.00); 1392	−6.00 (−16.75, 4.50); 1351
	0.1 (−0.5, 0.6)	0.4 (0.0, 0.9)	0.2 (−0.2, 0.7)	0.0 (−0.5, 0.5)	−0.1 (−0.7, 0.5)	−0.5 (−1.5, 0.4)
2–3 years	0.50 (−4.00, 5.50); 9898	2.50 (−1.00, 7.50); 1368	1.50 (−2.00, 6.00); 2906	0.00 (−4.00, 5.00); 2843	−1.00 (−6.00, 4.50); 1431	−4.00 (−12.50, 5.00); 1350
	0.0 (−0.4, 0.5)	0.2 (−0.1, 0.7)	0.1 (−0.2, 0.5)	0.0 (−0.4, 0.5)	−0.1 (−0.5, 0.4)	−0.4 (−1.1, 0.5)
1–2 years	ref.; 10,414	ref.; 1488	ref.; 3073	ref.; 2977	ref.; 1466	ref.; 1410
0–1 year	ref.; 11,834	ref.; 1677	ref.; 3311	ref.; 3265	ref.; 1673	ref.; 1908
Post-FM						
0–1 year	−2.50 (−9.00, 2.50); 10,761**	1.00 (−2.00, 5.50); 1545	−1.00 (−5.00, 3.00); 3046**	−3.00 (−8.00, 2.00); 2984**	−6.00 (−13.00, 0.50); 1508**	−15.50 (−31.00, −4.00); 1678**
	−0.2 (−0.8, 0.2)	0.1 (−0.2, 0.5)	−0.1 (−0.5, 0.3)	−0.3 (−0.7, 0.2)	−0.5 (−1.2, 0.0)	−1.4 (−2.8, −0.4)
1–2 years	−1.00 (−7.50, 5.00); 5300**	3.00 (−1.00, 8.50); 837	0.00 (−4.50, 5.00); 1700	−2.00 (−8.00, 4.00); 1445**	−6.00 (−12.00, 2.00); 650**	−14.50 (−28.50, −1.00); 668**
	−0.1 (−0.7, 0.5)	0.3 (−0.1, 0.8 )	0.0 (−0.4, 0.5)	−0.2 (−0.7, 0.4)	−0.5 (−1.1, 0.2)	−1.3 (−2.6, −0.1)
2+ years	−2.50 (−9.00, 3.50); 758**	−1.00 (−1.88, 6.50); 126	−1.50 (−6.00, 4.00); 283*	−4.50 (−10.50, 2.00); 193**	−5.0 0 (−15.00, 3.00); 77**	−21.00 (−34.00, −10.50); 79**
	−0.2 (−0.8, 0.3)	0.1 (−0.2, 0.6)	−0.1 (−0.5, 0.4)	−0.4 (−1.0, 0.2)	−0.5 (−1.4, 0.3)	−1.9 (−3.1, −1.0)

Data are median (IQR); *n*; median (IQR) results are dual reported in HbA_1c_ percentage units in rows below the main mmol/mol results

For post-exposure years, *p < 0.05 and **p < 0.01 for change from FM initiation (Wilcoxon signed-rank test *p* adjusted for multiple comparisons)

#### By age band

**Fig. 2:**
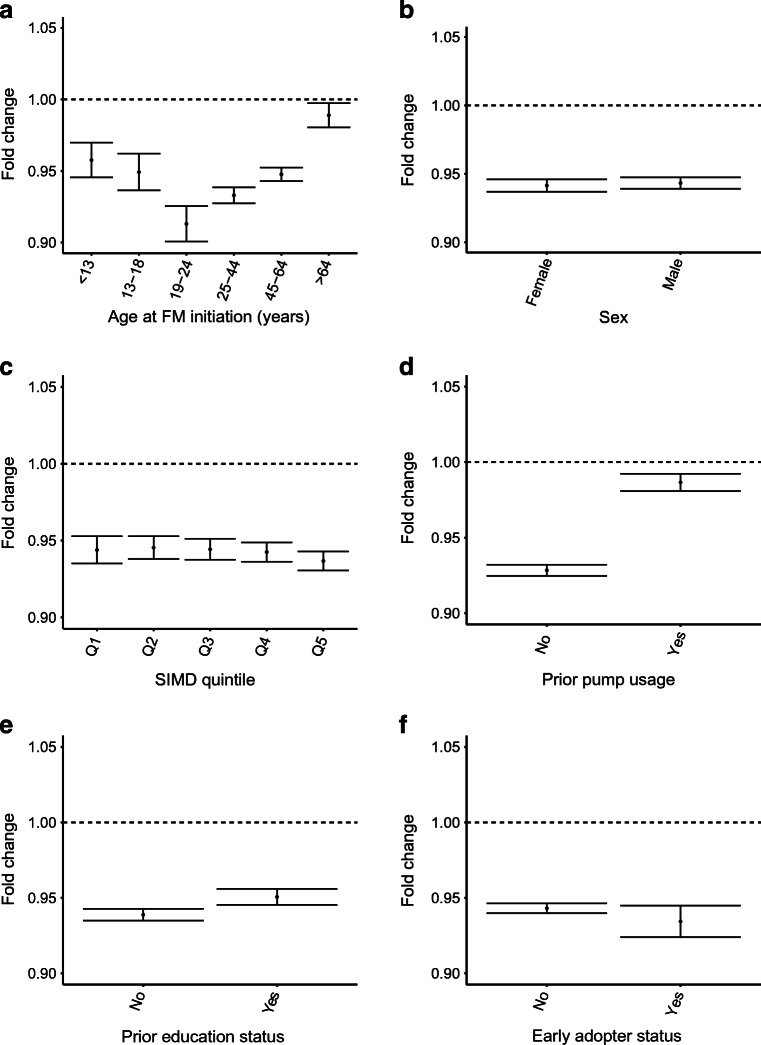
Estimated fold changes (95% CI) in HbA_1c_ within the first year post FM initiation, compared with pre-exposure levels, adjusted for pre-exposure trend, baseline HbA_1c_, age, sex and diabetes duration and stratified by age band at FM initiation (**a**), sex (**b**), SIMD quintile (**c**), prior pump usage (**d**), prior completed diabetes education programme status (**e**) and early adopter status (**f**)

FM initiation was associated with a reduction in HbA_1c_ in all age bands (Fig. 2a), with smaller estimated changes in the >64 years band. There was significant variation in HbA_1c_ at initiation by age (ESM Table 3), with HbA_1c_ being lowest in those aged <13 years and highest in those aged 19–24 years. As expected from this, reductions in HbA_1c_ were greatest among the 19–24 years age band. Among those aged 13–18 years, the median observed within-person change was 0.0 (−7.0, 7.0) mmol/mol (0.0 [−0.6, 0.6]%) (ESM Table 4). However, the modelled estimate, accounting for increase in HbA_1c_ pre-FM exposure, suggested a reduction compared with the counterfactual, with a 0.95 (95% CI 0.94, 0.96) fold change (ESM Table 5). Within any age band, in those with high HbA_1c_ (≥75 mmol/mol [9.0%]) at FM initiation, clear reductions were observed in HbA_1c_. These were most pronounced in children (<13 years), with a median (IQR) within-person fall of −30.5 (−50.0, −12.0) mmol/mol (−2.8 [−4.6, −1.1]%) (ESM Table 6). Allowing for differences in initial HbA_1c_, there was evidence of some variation in the fold change by age band (*p* for age group × FM interaction <0.01).

#### By sex

FM initiation was associated with a similar reduction in HbA_1c_ in men and women respectively (*p*_*interaction*_ = 0.18; Fig. 2b).

#### By SIMD

FM initiation was associated with a reduction in HbA_1c_ in all SIMD quintiles. The magnitude of reduction was similar across quintiles (*p*_*interaction*_ = 0.10; Fig. 2c), despite those from more-deprived quintiles presenting with higher baseline HbA_1c_ (ESM Tables 3, 7, 8).

#### By prior pump usage

FM initiation was associated with a reduction in HbA_1c_ regardless of prior pump use, although reductions were smaller in prior pump users (Fig. 2d and ESM Tables 9, 10), as expected from their lower HbA_1c_ at baseline compared with those with no prior pump use (ESM Table 3). However, when allowing for differences in baseline HbA_1c_, there was still evidence of some variation in effect by prior pump usage (*p* < 0.01 for FM × pump interaction).

#### By prior completed diabetes education programme

FM initiation was associated with a reduction in HbA_1c_ regardless of prior education status (Fig. 2e and ESM Tables 11, 12). Reductions were higher in those without completed education, while both groups had similar baseline HbA_1c_ (ESM Table 3).

#### By early adopter status

FM initiation was associated with similar reductions in HbA_1c_ regardless of early FM adoption (Fig. 2f and ESM Tables 13, 14).

### Changes in DKA rates

**Fig. 3:**
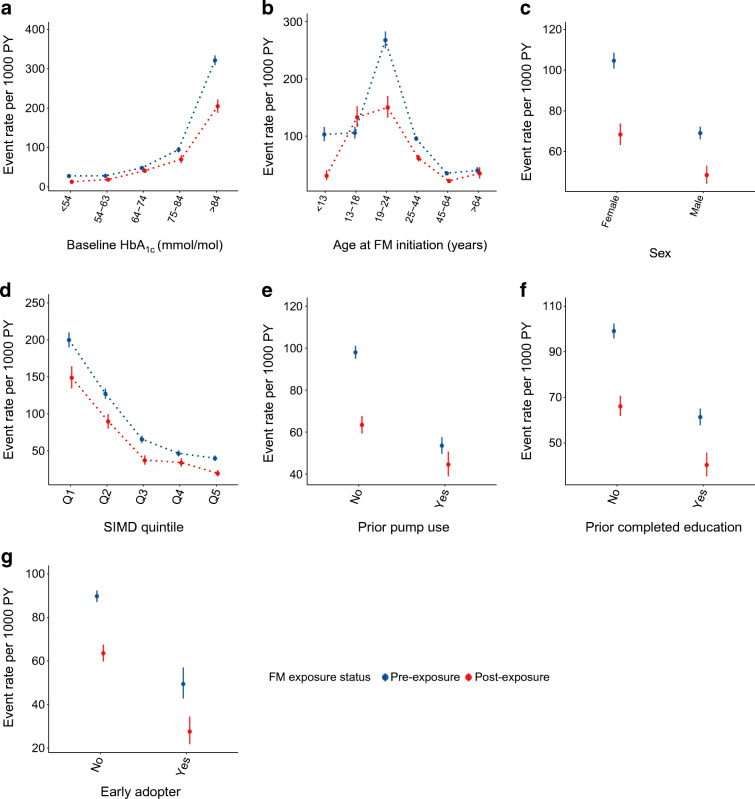
Crude DKA event rates (95% CI) in FM users, after and before FM initiation, stratified by baseline HbA_1c_ at initiation (**a**), age at FM initiation (**b**), sex (**c**), SIMD quintile (**d**), prior pump usage (**e**), prior completed diabetes education programme status (**f**) and early adopter status (**g**). PY, person-years

There were 53,046 person-years observable for DKA/SHH events before FM initiation and 19,001 afterwards. DKA rates pre-FM initiation varied considerably across the strata of interest (Fig. 3). Pre-FM rates were higher in those with high baseline HbA_1c_, young adults, those from more-deprived areas, those with no prior pump use, those with no completed diabetes education programme and in the non-early-adopters.

DKA rates significantly decreased overall after FM initiation (estimated rate ratio from the Bayesian models was 0.59 [95% CrI 0.53, 0.64], see Table 3). At the same time, rates across non-users decreased slightly but to a much lesser magnitude: crude rate ratio (95% CI) for post- vs pre-index time 0.90 (0.85, 0.96) vs 0.67 (0.63, 0.72) in users.

**Table 3:** DKA and SHH crude event rates and estimated rate ratio from adjusted Bayesian models

Event	Crude rate pre-FM^a^	Crude rate post-FM^a^	Rate ratio (95% CrI)
DKA			
Overall	86.8 (84.3, 89.3); 4604; 53,046.1	58.4 (55.0, 62.0); 1110; 19,000.8	0.59 (0.53, 0.64)
With prior DKA history	544.2 (528.6, 560.1); 4604; 8460.2	267.7 (249.3, 287.0); 789; 2947.5	0.44 (0.40, 0.49)
SHH			
Overall	19.2 (18.1, 20.4); 1020; 53,046.1	17.5 (15.6, 19.5); 332; 19,000.8	0.75 (0.62, 0.90)
With prior SHH history	312.2 (293.4, 332.0); 1020; 3266.8	106.7 (88.0, 128.2); 114; 1068.1	0.25 (0.20, 0.32)

^a^Data are presented as crude rate (95%CI); *n* events observed; *n* person-years observed

Crude DKA rates decreased in pre- vs posts-FM person-time among all subgroups examined (Fig. 3), apart from the adolescent group, where an increase was observed (Fig. 3b). All reductions were significant apart for those with prior pump use and those with baseline HbA_1c_ 64–74 mmol/mol (8.0–8.9%) (Fig. 3a,e).

The Bayesian models adjusting for prior trends showed reductions in all subgroups apart from those with prior pump use and those with baseline HbA_1c_ 54–63 mmol/mol (7.1–7.9%), where there was uncertainty around the result (ESM Fig. 3). Estimated reduction in rates was most marked among those with baseline HbA_1c_ ≥ 75 mmol/mol (9.0%) and those with HbA_1c_ < 54 mmol/mol (7.1%), though the credible interval was extremely wide in this subgroup due to the low number of events (ESM Fig. 3a).

Estimated reductions were most substantial in children (ESM Fig. 3b). Model results also indicated that, accounting for increase in DKA rate in pre-FM years, FM was associated with a reduction in DKA rate among adolescents, compared with the counterfactual. The magnitude of estimated reduction was higher in male vs female participants (ESM Fig. 3c), in those from least- vs most-deprived areas (ESM Fig. 3d) and in those without vs with prior pump use (ESM Fig. 3.e). Model results also suggested a higher reduction in those without vs with prior completed diabetes education programme (ESM Fig. 3f) and early adopters of FM (ESM Fig. 3g), although CrIs were wide and overlapped.

### Changes in SHH rates

SHH rates slightly decreased overall post-FM (Table 3) Among those with a prior SHH history, the crude event-rate was significantly lower during FM-exposed person-time. Bayesian model estimates supported this finding (estimated rate ratio 0.25 [95% CrI 0.20, 0.32], see Table 3). We did not have enough statistical power to analyse pre–post differences among those with starting HbA_1c_ < 54 mmol/mol (7.1%), who will probably have been prescribed FM due to recurrent hypoglycaemia. There were only 109 events observed pre-FM (crude rate 15.4 [12.7, 18.6] per 1000 person-years) and 26 post-FM (crude rate 9.3 [6.1, 13.7] per 1000 person-years).

## Discussion

This study showed that prevalence of FM use increased rapidly among individuals with type 1 diabetes in Scotland after FMs became free of charge but disparities remain across deprivation levels. FM initiation was associated with a significant decrease in HbA_1c_ overall among users. HbA_1c_ reductions were most pronounced in those with high baseline HbA_1c_. HbA_1c_ reductions occurred in all SIMD quintiles and age groups, and regardless of sex, prior pump use, early adopter status or prior completed diabetes education programme. FM use was associated with marked reductions in DKA overall and generally within all subgroups examined. FM initiation was also associated with a decrease in SHH among those with a prior history of SHH.

To our knowledge, our large nationwide study is the first to examine disparities in the prevalence of FM use in Scotland. We have confirmed and extended previous glycaemic outcome findings of small-scale studies in Scotland [6, 22] by providing generalisable results. We have also augmented the scope of recent large-scale studies [13, 14] by extensively exploring variations in HbA_1c_ and DKA outcomes following the initiation of FM use across sociodemographic strata, which has not been done before and provides novel information crucial to clinical practice.

Efforts made by the Scottish Government, clinical teams, charities such as Diabetes UK, and people with diabetes to widen the usage of FMs in Scotland have been successful, with a tenfold increase in use over the past couple of years. However, the gap between most- and least-deprived quintiles persists, although it is smaller than the 4% vs 60% observed in the most- vs least-deprived quintiles in an Edinburgh diabetes centre in 2017 prior to NHS funding [3]. This gap highlights the existence of healthcare inequalities in access to technology. The extent to which this relates to user preference or to failure of the devices being recommended by clinicians is unclear. Prevalence of use is highest among the paediatric population but gaps across deprivation levels exist even in this group.

Our overall findings on HbA_1c_ reductions are in keeping with previous findings such as those from a single-centre Edinburgh study (−4 mmol/mol [−0.4%]) [22], meta-analyses performed on FM and HbA_1c_, mean −4.5 mmol/mol [−0.4%] in uncontrolled studies [7], and a registry-study from the Netherlands (mean −3.3 mmol/mol [−0.3%]) [11]. Less than half of the FM users were followed-up for more than 1 year post-initiation, therefore more longitudinal follow-up is needed to establish the long-term persistence of the improvements in HbA_1c_.

Only a few studies have looked at FM use and DKA so far. Our findings regarding DKA overall are in keeping with those of other nationwide studies regarding DKA hospitalisation rates [9, 14]. In a French nationwide database, Roussel et al. [14] reported that DKA hospitalisation rates fell by 56.2% in the year after vs before FM initiation. This reduction is beneficial in terms of individuals’ wellbeing and reductions in healthcare costs, as DKA is expensive to treat [23].

Stratified analyses of DKA rates following FM initiation are lacking in the literature. The variations in HbA_1c_ changes from baseline across starting HbA_1c_ were in keeping with those reported in previous studies: slight increase among those with optimally controlled baseline HbA_1c_ [6, 24]; and substantial decrease among those with high baseline HbA_1c_ [6, 7, 10, 13, 22]. We also found that reductions in DKA rates post- vs pre-FM were most marked in those with high baseline HbA_1c_. These improvements are extremely promising and likely to translate into a reduction in healthcare costs as those with high HbA_1c_ levels are most at risk of complications [25].

We found that FM use was associated with improvements in HbA_1c_ in all SIMD quintiles, showing that this technology benefits all, including those from more-deprived areas. Tsur et al. [9] also reported significant improvements in HbA_1c_ among those with lower socioeconomic status. Although the magnitude of reduction in DKA rates was higher among those from least-deprived areas, there were marked improvements in all SIMD quintiles. Unequal distribution of, or access to, this technology may further widen existing inequalities in healthcare, especially since those from more-deprived areas have historically higher HbA_1c_ [26] and thus stand to benefit most from FM.

Existing paediatric studies have had small sample sizes [7, 8] with heterogeneous findings. For example, Campbell et al. [27] reported a significant decrease in HbA_1c_ among children aged 4–17 years, while Messaaoui et al. [28] reported no change in HbA_1c_ among their sample of children/young people aged 4–20 years. In our study, HbA_1c_ reduction appeared to be smaller among the paediatric group, although this was expected considering the well-controlled baseline HbA_1c_. Conversely, reduction in DKA rates was substantial in children. Among those with high baseline HbA_1c_, marked reductions in HbA_1c_ were observed in all age groups.

Despite minimal observed reduction in HbA_1c_ and observed increase in crude DKA rate among adolescents, model results accounting for prior trends suggested improvement in both areas. Longer post-FM follow-up is needed among adolescents to better understand how or whether FM use mitigates the usual deterioration in HbA_1c_ among this age group. It is also important to consider factors other than blood glucose outcomes when evaluating the benefits of FM in this group, such as quality of life. Indeed, qualitative studies [29, 30] have suggested such improvements in this demographic. Al Hayek et al. [31] also found a significant reduction in diabetes distress in a sample of 187 adolescents. However, we do not have access to such data and additional work needs to be done to examine whether FM usage among adolescents could be improved further.

The smaller reductions observed among those with prior pump use was consistent with their lower baseline HbA_1c_, and was in keeping with other findings [9]. Individuals using insulin pumps in Scotland attend a structured education programme prior to pump initiation and receive substantial input from diabetes support services. Therefore, gains in terms of HbA_1c_ are expected to be marginal in this group. The non-significant decrease in DKA is likely due to significant improvements already occurring following pump initiation [32]. Improvements in this group are expected in terms of quality of life or hypoglycaemia but we did not possess data to assess this.

DKA and HbA_1c_ improved regardless of completion of a diabetes education programme but individual education levels were not available to assess their influence on outcomes.

Interestingly, disparities in DKA rates between strata before FM initiation generally persisted even after the post-FM reductions. This highlights the need to better understand drivers of elevated DKA rates. Indeed, O’Reilly et al. [33] showed that factors beyond structured education, use of pump and HbA_1c_ likely contributed to elevated rates among most-deprived quintiles.

Our findings suggest that FM use is associated with a reduction in SHH among those at risk of this complication. Results on FM usage and hypoglycaemia in the literature vary. The IMPACT study [4] showed a reduction in hypoglycaemia in those with well-controlled HbA_1c_. Observational studies reported a significant decrease in severe hypoglycaemia [5, 9, 13, 14], while Campbell et al. [27] found time in hypoglycaemia to be unaffected in their paediatric sample. Differences in results are likely due to a combination of differing hypoglycaemia definitions and cohort characteristics/behaviour. It is nonetheless important to understand whether there is any over-adjustment of insulin dose following readings of FM data.

### Strengths and limitations

Our study is one of the largest contemporary real-world-setting studies examining the association of FM initiation with glycaemic outcomes combining data from nationwide electronic health records with extensive subgroup analyses, in particular filling a gap in the literature with regards to FM use and DKA. Using data from all individuals with type 1 diabetes in Scotland, we were able to capture current disparities in usage in the country and had enough power to explore a large number of sociodemographic group-specific outcomes.

For comparison, a recent large-scale UK-based voluntary audit [10] possessed post-FM follow-up HbA_1c_ measures for only one-third of the users included (3182 out of 9968), while recent national Swedish and French studies [13, 14] did not examine variations across sociodemographic groups.

We were limited in our analyses of hypoglycaemia by only being able to analyse hospital admissions, which represent a tiny fraction of hypoglycaemic events [34]. We did not have access to granular glucose data from the Libre devices; this would have allowed better understanding of glycaemic variability and analysis of hypoglycaemia with more precision. Our study suffers from the usual biases linked to observational studies, such as unmeasured confounding or measurement error. Since this study was observational, observed changes were not attributable to FM use in the clear-cut manner of an RCT. However, timing of changes and crude comparisons to non-users support the findings in relation to FM initiation.

Since the end of our study, newer FM models such as the Libre 2 have become available (since January 2021). Our findings pertaining to marked improvements even with first-generation Libre devices herald positive outcomes with more updated Libre versions.

Due to the criteria of eligibility for FM use, our results might not be generalisable to all those with type 1 diabetes. These criteria are less restrictive than eligibility to insulin pumps, which were also found to be associated with improved glycaemic outcomes among people with type 1 diabetes in Scotland [32]. It is nonetheless crucial to understand the determinants of good response to FMs to optimise a more widespread roll-out. For example, Riveline et al. [35], among others, showed that scanning frequency is associated with better glycaemic outcomes; however, we did not have access to such data.

### Conclusions

Flash glucose monitoring use in Scotland has been associated with clinically important improvements in HbA_1c_, especially in individuals with high baseline HbA_1c_ who have the most to gain in reducing the risk of diabetes complications. Historically, reducing rates of DKA has proven to be an extremely difficult task and uptake of effective interventions (such as structured education) has often been relatively low. The striking reduction in DKA across the sociodemographic spectrum following FM use is of major clinical importance. More research is needed to better understand how to increase the uptake of FM use and the drivers and features of its effect in order to tighten the existing socioeconomic gaps. Results will need to be updated when longer-term follow-up is available and to keep pace with newer technologies and systems such as newer Libre models, DIY closed-loop systems or officially licensed hybrid-loop systems.

## Supplementary Information

ESM 1 (PDF 653 kb)

## Data Availability

We do not have governance permissions to share individual-level data on which these analyses were conducted. However, bona fide researchers can apply to the Scottish Public Benefits and Privacy Protection Committee for access to these data. This research was conducted with approval from the Public Benefit Privacy Protection Panel (PBPP ref. 1617- 0147). All datasets were de-identified before analysis.

## Abbreviations

CGM: Continuous glucose monitoring
CrI: Credible interval
DKA: Diabetic ketoacidosis
FM: Flash monitor
NHS: National Health Service
SCI-DC: Scottish Care Information - Diabetes Collaboration
SHH: Severe hospitalised hypoglycaemia
SIMD: Scottish Index of Multiple Deprivation
