# Supplementary material for: Flash monitor initiation is associated with improvements in HbA1c levels and DKA rates among people with type 1 diabetes in Scotland: a retrospective nationwide observational study
Source: Diabetologia. 2021 Oct 7;65(1):159–72. doi: 10.1007/s00125-021-05578-1 (PMC8660764; doi:10.1007/s00125-021-05578-1)
Supplement: Supplementary file 1 — (PDF 653 kb) [file 125_2021_5578_MOESM1_ESM.pdf]

Supplementary Material for Flash monitor initiation is associated with improvements in HbA$_{1c}$ levels and DKA rates among people with type 1 Diabetes in Scotland: a retrospective nationwide observational study

A. Jeyam F.W. Gibb J.A. McKnight J.E. O'Reilly T.M. Caparrotta A. Höhn
S.J. McGurnaghan L.A.K. Blackbourn S. Hatam B. Kennon R. McCrimmon
G. Leesel S. Philip N. Sattar P. M. McKeigue H. M. Colhoun
On behalf of the SDRN Epi Group

Methods

Exposure, outcomes and covariates

Diabetic ketoacidosis (DKA) events were defined as any hospital admission or death, involving an ICD10 diagnosis code of E10.1, E11.1, E12.1, E13.1 or E14.1 at any level of the reasons for admission or causes of death.

Severe hypoglycaemic (SHH) events were defined as any hospital admission or death involving an ICD10 diagnosis code of E15, E16.0, E16.1, E16.2 at any level of the reasons for admission or causes of death.

Statistical analyses

HbA$_{1c}$

Since the distribution of HbA$_{1c}$ was skewed,HbA$_{1c}$ was log-transformed. We used linear mixed models with log-transformed HbA$_{1c}$ as the outcome. Fixed effects included were age at flash monitor (FM) initiation, diabetes duration at FM initiation, sex, log(baseline HbA$_{1c}$), time (in years and centered on FM initiation such date of FM initiation=0) and categorical time of FM-usage (years, reference category=no FM usage). The model also included random effects on the individuals: random intercept and slope on time. The within-individual error-correlation structure was specified as a CAR1 autoregressive structure to allow for exponentially decayed correlation in measurements the further apart they are in time.

We formally tested the significance of interaction terms using fully adjusted versions of the mixed models described previously. Fixed effects included were: age, sex, SIMD, diabetes duration, baseline HbA$_{1c}$, FM-exposure time and the interaction term (time of FM-use)*group of interest. Significance of the interaction term was assessed using Likelihood Ratio Tests comparing models with and without the interaction term. For this purpose, models were fitted using Maximum Likelihood (ML) so as to appropriately compare models with different fixed effects.

Sensitivity analyses

To account for any trends occuring over the same calendar-time as FM-initiation, we randomly selected for each FM user from the population under study, an individual matched in age band and baseline HbA1c band, who was not using any device (CSII/FM/CGM) at the date of FM-initiation in the user and for at least six months thereafter and assigning the user's FM start date as their index date. Non-users' person-time were censored as soon as they started using any device.

Results

ESM Table 1: Characteristics of FM users at baseline (defined as the two-year window prior to FM initiation), and of the matched non-users

Variable	FM users (N=12,256)		Matched non-users (N=11,682)	
Variable	N(%) or Median (IQR)	Missing (%)	N(%) or Median (IQR)	Missing (%)
Sex: Female	6073 (49.6)	0.0	5751 (49.2)	0.0
Age at initiation	37.9 (24.2, 52.6)	0.0	39.3 (25.6, 54.1)	0.0
Diabetes duration at initiation	17.5 (6.9, 29.3)	0.0	17.8 (7.4, 29.0)	0.0
Prior Pump/CGM usage	2867 (23.4)	0.0	-	0.0
HbA_{1c} band at baseline (mmol/mol)		1.2		4.2
<54 [7.1%]	1711 (14.1)		1606 (14.4)	
54-63 [7.1-7.9%]	3381 (27.9)		2814 (25.2)	
64-74 [8.0-8.9%]	3345 (27.6)		3019 (27.0)	
75-84 [9.1-9.8%]	1717 (14.2)		1708 (15.3)	
>84 [9.8%]	1956 (16.2)		2039 (18.2)	
BMI (kg/m^2)	25.4 (22.2, 29.0)	3.7	25.8 (22.4, 29.6)	10.0
Total cholesterol (mmol/l)	4.6 (4.0, 5.2)	16.9	4.6 (4.0, 5.3)	21.5
Systolic blood pressure (mmHg)	128.0 (119.0, 138.0)	8.9	128.5 (119.0, 138.0)	11.0
Diastolic blood pressure (mmHg)	76.0 (70.0, 81.0)	8.9	75.0 (70.0, 80.5)	11.0
CKDEPI- eGFR (mL/min/1.73m^2)		10.9		13.7
<15	109 (1.0)		157 (1.6)	
15-30	57 (0.5)		94 (0.9)	
30-60	498 (4.6)		537 (5.3)	
60-90	2572 (23.5)		2097 (20.8)	
≥90	7688 (70.4)		7198 (71.4)	
Albuminuric status: micro/macro albuminuria	2185 (23.9)	25.3	2012 (26.7)	35.6
SIMD Quintile		0.0		7.3
Q1 (most deprived)	1778 (15.5)		2589 (23.9)	
Q2	2354 (20.5)		2340 (21.6)	
Q3	2324 (20.2)		2201 (20.3)	
Q4	2493 (21.7)		2002 (18.5)	
Q5 (least deprived)	2548 (22.2)		1698 (15.7)	
Ever prior DKA admission in the last 5 years	2026 (16.5)	0.1	2047 (17.5)	0.1
Ever prior Hypo admission in the last 5 years	712 (5.8)	0.1	646 (5.5)	0.1

ESM Table 2: Estimated fold-change (95 % CI) in HbA_{1c} yearly pre-exposure and for FM-exposed time compared to the counterfactual- overall and stratified by baseline HbA_{1c} band- from mixed models adjusting for sex, baseline HbA_{1c}, age and diabetes duration at FM initiation

Variable	Overall	Baseline HbA_{1c} band				
		<54 mmol/mol [7.1%]	54-63 mmol/mol [7.1-7.9%]	64-74 mmol/mol [8.0-8.9%]	75-84 mmol/mol [9.0-9.8%]	>84 mmol/mol
Time effect (years)	0.99 (0.99; 0.99)	0.96 (0.96; 0.96)	0.98 (0.98; 0.98)	0.99 (0.99; 1.00)	1.00 (1.00; 1.01)	1.01 (1.01; 1.02)
**FM usage (ref=no FM usage)**						
0-1 year	0.94 (0.94; 0.95)	1.08 (1.07; 1.09)	0.99 (0.99; 1.00)	0.95 (0.95; 0.96)	0.91 (0.90; 0.92)	0.77 (0.76; 0.78)
1-2 years	0.98 (0.98; 0.99)	1.18 (1.16; 1.19)	1.03 (1.03; 1.04)	0.98 (0.98; 0.99)	0.93 (0.91; 0.94)	0.79 (0.78; 0.81)
2 or more years	0.99 (0.98; 1.00)	1.22 (1.18; 1.25)	1.06 (1.05; 1.08)	0.98 (0.96; 1.00)	0.91 (0.88; 0.94)	0.75 (0.72; 0.78)
Number of observations	63330	8952	18526	17793	8993	9066
Number of individuals	11353	1621	3173	3114	1614	1831

ESM Table 3: Baseline HbA_{1c} distribution (mmol/mol) - Median (IQR) - across strata of interest. Data are dual reported in HbA_{1c} percentage units.

Strata		Baseline HbA1c distribution (mmol/mol)	N
Age-band at FM initiation	<13	61.5 (55.5, 70.0)<br>7.8 (7.2, 8.6)%	1063
	13-18	66.0 (58.0, 80.0)<br>8.2 (7.5, 9.5)%	1028
	19-24	72.0 (61.0, 88.0)<br>8.7 (7.7, 10.2)%	1137
	25-44	67.0 (58.0, 79.5)<br>8.3 (7.5, 9.4)%	4323
	45-64	66.5 (58.0, 76.5)<br>8.2 (7.5, 9.1)%	3775
	>64	64.0 (57.0, 72.0)<br>8.0 (7.4, 8.7)%	930
SIMD quintile	Q1 (most deprived)	71.0 (61.0, 85.0)<br>8.6 (7.7, 9.9)%	1778
	Q2	69.0 (60.0, 80.0)<br>8.5 (7.6, 9.5)%	2354
	Q3	66.0 (58.0, 78.0)<br>8.2 (7.5, 9.3)%	2324
	Q4	65.0 (57.0, 75.5)<br>8.1 (7.4, 9.1)%	2493
	Q5 (least deprived)	63.0 (56.0, 72.0)<br>7.9 (7.3, 8.7)%	2548
Sex	Female	67.0 (58.0, 78.0)<br>8.3 (7.5, 9.3)%	6073
	Male	66.0 (58.0, 77.0)<br>8.2 (7.5, 9.2)%	6183
Prior/current pump use	No	68.0 (59.0, 80.0)<br>8.4 (7.5, 9.5)%	9389
	Yes	61.5 (55.0, 69.0)<br>7.8 (7.2, 8.5)%	2867
Prior CMG use	No	66.0 (58.0, 78.0)<br>8.2 (7.5, 9.3)%	12196
	Yes	60.5 (53.0, 67.5)<br>7.7 (7.0, 8.3)%	60
Prior completed education	No	66.0 (58.0, 78.0)<br>8.2 (7.5, 9.3)%	8587
	Yes	66.0 (58.0, 76.5)<br>8.2 (7.5, 9.1)%	3669
Early adopter	No	66.5 (58.0, 78.0)<br>8.2 (7.5, 9.3)%	11303
	Yes	64.0 (56.5, 74.0)<br>8.0 (7.3, 8.9)%	953

ESM Table 4: Absolute within-person differences in HbA_{1c} (mmol/mol), with respect to baseline, over time from FM initiation (years), stratified by age band at FM initiation - N and median (IQR). Data are dual reported in HbA_{1c} percentage units.

Time from FM initiation (yrs)	Age <13 y.o.	Age 13-18 y.o.	Age 19-24 y.o.	Age 25-44 y.o.	Age 45-64 y.o.	Age >64 y.o.
**Pre-FM**						
4-5 yrs bef.	2.00(-3.00,8.00)|282<br>0.2(-0.3,0.7)%	-2.50(-11.00,5.00)|611<br>-0.2(-1.0,0.5)%	0.50(-9.00,10.50)|803<br>0.0(-0.8,1.0)%	2.00(-5.00,10.00)|3260<br>0.2(-0.5,0.9)%	1.00(-4.00,7.00)|3259<br>0.1(-0.4,0.6)%	0.88(-4.00,5.50)|866<br>0.1(-0.4,0.5)%
3-4 yrs bef.	1.00(-4.00,6.00)|402<br>0.1(-0.4,0.5)%	-2.50(-11.50,4.50)|707<br>-0.2(-1.1,0.4)%	1.00(-8.00,10.00)|834<br>0.1(-0.7,0.9)%	1.00(-5.00,8.50)|3389<br>0.1(-0.5,0.8)%	1.00(-4.00,6.00)|3308<br>0.1(-0.4,0.5)%	1.00(-3.62,5.00)|880<br>0.1(-0.3,0.5)%
2-3 yrs bef.	1.00(-3.00,5.50)|545<br>0.1(-0.3,0.5)%	-1.50(-8.00,4.00)|785<br>-0.1(-0.7,0.4)%	1.00(-5.00,8.25)|853<br>0.1(-0.5,0.8)%	1.00(-4.00,6.50)|3486<br>0.1(-0.4,0.6)%	0.50(-3.00,5.00)|3357<br>0.0(-0.3,0.5)%	0.50(-3.00,4.00)|872<br>0.0(-0.3,0.4)%
1-2 yrs bef.	ref.|710	ref.|862	ref.|904	ref.|3627	ref.|3422	ref.|889
0-1 yr bef.	ref.|1042	ref.|1012	ref.|1071	ref.|4129	ref.|3667	ref.|913
**Post-FM**						
0-1 yr aft.	-1.50(-7.50,3.00)|1005**<br>-0.1(-0.7,0.3)%	0.00(-7.00,7.00)|968-<br>0.0(-0.6,0.6)%	-4.50(-14.00,2.50)|927**<br>-0.4(-1.3,0.2)%	-4.00(-11.00,1.50)|3682**<br>-0.4(-1.0,0.1)%	-3.00(-8.50,1.50)|3341**<br>-0.3(-0.8,0.1)%	-0.88(-5.00,3.50)|838**<br>-0.1(-0.5,0.3)%
1-2 yrs aft.	1.00(-5.00,6.00)|618-<br>0.1(-0.5,0.5)%	4.00(-3.50,12.00)|533-<br>0.4(-0.3,1.1)%	-4.00(-14.00,4.50)|403**<br>-0.4(-1.3,0.4)%	-2.00(-9.50,4.00)|1767**<br>-0.2(-0.9,0.4)%	-2.50(-8.00,3.00)|1556**<br>-0.2(-0.7,0.3)%	0.50(-4.00,5.00)|423-<br>0.0(-0.4,0.5)%
2+ yrs aft.	0.00(-4.50,4.00)|106-<br>0.0(-0.4,0.4)%	5.00(-5.00,11.25)|82-<br>0.5(-0.5,1.0)%	-9.00(-20.50,4.50)|54**<br>-0.8(-1.9,0.4)%	-3.00(-10.62,2.50)|271**<br>-0.3(-1.0,0.2)%	-5.00(-9.50,0.12)|187**<br>-0.5(-0.9,0.0)%	-2.25(-7.62,3.12)|58-<br>-0.2(-0.7,0.3)%

Note:

For post-exposure years, significance of Wilcoxon signed-rank test p adjusted for multiple comparisons denoted by ** (p<0.01), * ($0.01 \leq p<0.05$) or - (NS)

ESM Table 5: Estimated fold-change (95 % CI) in HbA_{1c} yearly pre-exposure and for FM-exposed time compared to the counterfactual- stratified by age at FM initiation from mixed models adjusting for sex, baseline HbA_{1c}, age and diabetes duration at FM initiation

Variable	<13 y.o.	13-18 y.o.	19-24 y.o.	25-44 y.o.	45-64 y.o.	>64 y.o.
Time effect (years)	0.97 (0.96; 0.97)	1.01 (1.01; 1.02)	0.99 (0.99; 1.00)	0.99 (0.99; 0.99)	0.99 (0.99; 0.99)	1.00 (0.99; 1.00)
**FM usage (ref=no FM usage)**						
0-1 year	0.96 (0.95; 0.97)	0.95 (0.94; 0.96)	0.91 (0.90; 0.93)	0.93 (0.93; 0.94)	0.95 (0.94; 0.95)	0.99 (0.98; 1.00)
1-2 years	1.05 (1.03; 1.06)	1.00 (0.98; 1.02)	0.94 (0.92; 0.96)	0.97 (0.96; 0.98)	0.97 (0.97; 0.98)	1.03 (1.01; 1.04)
2 or more years	1.10 (1.06; 1.14)	1.01 (0.97; 1.05)	0.92 (0.88; 0.97)	0.98 (0.96; 1.00)	0.97 (0.96; 0.99)	1.03 (1.00; 1.06)
Number of observations	4730	5562	5738	21762	20343	5195
Number of individuals	1042	1013	1086	3928	3451	833

ESM Table 6: Absolute within-person changes from baseline in HbA_{1c} (mmol/mol) following FM initiation - median (IQR)|N - by age band * baseline HbA_{1c} category. Data are dual reported in HbA_{1c} percentage units.

<table>
<tr><th></th><th><13y.</th><th>13-18y.</th><th>19-24y.</th><th>25-44y.</th><th>45-64y.</th><th>>64</th></tr>
<tr><td><58 mmol/mol [7.5%]</td><td></td><td></td><td></td><td></td><td></td><td></td></tr>
<tr><td>0-1 yr aft.</td><td>1.00(-2.00,4.00)|341
0.09(-0.18,0.37)%</td><td>2.25(-1.00,7.50)|238
0.21(-0.09,0.69)%</td><td>1.50(-3.00,6.00)|188
0.14(-0.27,0.55)%</td><td>0.00(-3.50,4.50)|894
0.00(-0.32,0.41)%</td><td>0.00(-3.50,4.00)|776
0.00(-0.32,0.37)%</td><td>0.50(-2.00,4.25)|231
0.05(-0.18,0.39)%</td></tr>
<tr><td>1-2 yrs aft.</td><td>3.00(-1.00,7.50)|239
0.27(-0.09,0.69)%</td><td>5.50(0.00,11.25)|159
0.50(0.00,1.03)%</td><td>3.00(-3.00,8.00)|89
0.27(-0.27,0.73)%</td><td>2.00(-3.00,6.25)|479
0.18(-0.27,0.57)%</td><td>1.00(-3.00,5.50)|389
0.09(-0.27,0.50)%</td><td>3.00(-1.50,6.50)|121
0.27(-0.14,0.59)%</td></tr>
<tr><td>2+ yrs aft.</td><td>0.00(-3.62,4.12)|47
0.00(-0.33,0.38)%</td><td>7.00(2.25,11.75)|21
0.64(0.21,1.08)%</td><td>5.50(-3.00,11.00)|13
0.50(-0.27,1.01)%</td><td>0.00(-3.00,6.50)|86
0.00(-0.27,0.59)%</td><td>-1.00(-5.00,4.00)|42
-0.09(-0.46,0.37)%</td><td>6.50(1.00,11.00)|16
0.59(0.09,1.01)%</td></tr>
<tr><td>58-75 mmol/mol [7.5-9.0%]</td><td></td><td></td><td></td><td></td><td></td><td></td></tr>
<tr><td>0-1 yr aft.</td><td>-1.00(-6.00,2.50)|491
-0.09(-0.55,0.23)%</td><td>1.00(-5.00,7.50)|421
0.09(-0.46,0.69)%</td><td>-3.00(-9.00,2.00)|335
-0.27(-0.82,0.18)%</td><td>-3.50(-8.50,1.00)|1598
-0.32(-0.78,0.09)%</td><td>-3.00(-7.00,1.00)|1607
-0.27(-0.64,0.09)%</td><td>-0.50(-5.00,3.00)|455
-0.05(-0.46,0.27)%</td></tr>
<tr><td>1-2 yrs aft.</td><td>0.75(-4.00,5.50)|296
0.07(-0.37,0.50)%</td><td>4.50(-3.00,12.31)|248
0.41(-0.27,1.13)%</td><td>-2.25(-11.50,5.00)|140
-0.21(-1.05,0.46)%</td><td>-2.50(-8.00,3.00)|796
-0.23(-0.73,0.27)%</td><td>-2.50(-7.00,2.62)|792
-0.23(-0.64,0.24)%</td><td>-10.25(-13.75,0.00)|<10
0.00(-0.45,0.37)%</td></tr>
<tr><td>2+ yrs aft.</td><td>1.25(-4.62,4.62)|46
0.11(-0.42,0.42)%</td><td>6.00(-5.00,12.50)|46
0.55(-0.46,1.14)%</td><td>-5.00(-13.00,4.00)|17
-0.46(-1.19,0.37)%</td><td>-4.50(-11.00,1.00)|125
-0.41(-1.01,0.09)%</td><td>-5.00(-8.50,-1.00)|109
-0.46(-0.78,-0.09)%</td><td>-4.00(-7.50,2.00)|34
-0.37(-0.69,0.18)%</td></tr>
<tr><td>≥75 mmol/mol [9.0%]</td><td></td><td></td><td></td><td></td><td></td><td></td></tr>
<tr><td>0-1 yr aft.</td><td>-30.50(-50.00,-12.00)|173
-2.79(-4.58,-1.10)%</td><td>-7.00(-26.50,5.00)|309
-0.64(-2.42,0.46)%</td><td>-12.00(-25.00,-1.00)|404
-1.10(-2.29,-0.09)%</td><td>-11.00(-21.50,-2.50)|1190
-1.01(-1.97,-0.23)%</td><td>-8.50(-16.00,-1.00)|958
-0.78(-1.46,-0.09)%</td><td>-4.75(-12.00,1.00)|152
-0.43(-1.10,0.09)%</td></tr>
<tr><td>1-2 yrs aft.</td><td>-19.50(-46.75,-4.50)|83
-1.78(-4.28,-0.41)%</td><td>-3.88(-14.38,11.00)|126
-0.35(-1.32,1.01)%</td><td>-12.00(-23.00,-1.12)|174
-1.10(-2.10,-0.10)%</td><td>-9.50(-21.12,0.12)|492
-0.87(-1.93,0.01)%</td><td>-8.00(-16.00,0.00)|375
-0.73(-1.46,0.00)%</td><td>-3.25(-9.00,3.62)|68
-0.30(-0.82,0.33)%</td></tr>
<tr><td>2+ yrs aft.</td><td>-10.00(-21.00,1.00)|13
-0.91(-1.92,0.09)%</td><td>-8.50(-29.00,1.50)|15
-0.78(-2.65,0.14)%</td><td>-20.50(-32.25,-10.50)|24
-1.88(-2.95,-0.96)%</td><td>-13.00(-28.00,1.00)|60
-1.19(-2.56,0.09)%</td><td>-15.00(-22.88,-4.25)|36
-1.37(-2.09,-0.39)%</td><td>-10.25(-13.75,0.00)|8
-0.94(-1.26,0.00)%</td></tr>
</table>

ESM Table 7: Absolute within-person differences in HbA_{1c} (mmol/mol) with respect to baseline over time from FM initiation (years), overall and stratified by SIMD quintile - Median (IQR)|N. Data are dual reported in HbA_{1c} percentage units.

<table>
<tr><th>Time from FM initiation (yrs)</th><th>Q1</th><th>Q2</th><th>Q3</th><th>Q4</th><th>Q5</th></tr>
<tr><td>Pre-FM</td><td></td><td></td><td></td><td></td><td></td></tr>
<tr><td>4-5 yrs bef.</td><td>1.00(-6.50,10.00)|1271
0.1(-0.6,0.9)%</td><td>1.00(-6.00,8.50)|1700
0.1(-0.5,0.8)%</td><td>1.00(-5.50,8.00)|1673
0.1(-0.5,0.7)%</td><td>1.00(-5.00,7.50)|1855
0.1(-0.5,0.7)%</td><td>1.00(-4.00,6.50)|1873
0.1(-0.4,0.6)%</td></tr>
<tr><td>3-4 yrs bef.</td><td>1.00(-6.50,8.00)|1323
0.1(-0.6,0.7)%</td><td>1.00(-6.00,7.00)|1782
0.1(-0.5,0.6)%</td><td>1.00(-4.50,7.00)|1785
0.1(-0.4,0.6)%</td><td>1.00(-5.00,6.00)|1921
0.1(-0.5,0.5)%</td><td>0.50(-4.50,6.00)|1984
0.0(-0.4,0.5)%</td></tr>
<tr><td>2-3 yrs bef.</td><td>1.00(-5.00,6.00)|1385
0.1(-0.5,0.5)%</td><td>0.50(-4.50,6.00)|1866
0.0(-0.4,0.5)%</td><td>0.50(-4.00,5.50)|1864
0.0(-0.4,0.5)%</td><td>0.50(-4.00,6.00)|2019
0.0(-0.4,0.5)%</td><td>0.50(-3.50,5.00)|2061
0.0(-0.3,0.5)%</td></tr>
<tr><td>1-2 yrs bef.</td><td>ref.|1471</td><td>ref.|1966</td><td>ref.|1969</td><td>ref.|2101</td><td>ref.|2187</td></tr>
<tr><td>0-1 yr bef.</td><td>ref.|1716</td><td>ref.|2274</td><td>ref.|2242</td><td>ref.|2400</td><td>ref.|2455</td></tr>
<tr><td>Post-FM</td><td></td><td></td><td></td><td></td><td></td></tr>
<tr><td>0-1 yr aft.</td><td>-2.50(-10.00,4.00)|1528**
-0.2(-0.9,0.4)%</td><td>-2.50(-9.50,3.00)|2068**
-0.2(-0.9,0.3)%</td><td>-2.50(-9.00,2.00)|2019**
-0.2(-0.8,0.2)%</td><td>-3.00(-9.00,2.00)|2194**
-0.3(-0.8,0.2)%</td><td>-3.00(-8.50,1.50)|2272**
-0.3(-0.8,0.1)%</td></tr>
<tr><td>1-2 yrs aft.</td><td>-0.50(-8.00,6.00)|718-
0.0(-0.7,0.5)%</td><td>-0.50(-8.00,6.00)|1012*
0.0(-0.7,0.5)%</td><td>-1.50(-8.00,5.00)|996**
-0.1(-0.7,0.5)%</td><td>-1.50(-8.00,4.00)|1116**
-0.1(-0.7,0.4)%</td><td>-1.50(-7.00,4.00)|1257**
-0.1(-0.6,0.4)%</td></tr>
<tr><td>2+ yrs aft.</td><td>-4.50(-11.00,2.00)|70**
-0.4(-1.0,0.2)%</td><td>-2.50(-10.00,3.75)|106**
-0.2(-0.9,0.3)%</td><td>-2.00(-8.00,3.00)|131**
-0.2(-0.7,0.3)%</td><td>-3.00(-9.50,5.00)|167**
-0.3(-0.9,0.5)%</td><td>-2.00(-8.00,4.00)|272**
-0.2(-0.7,0.4)%</td></tr>
</table>

Note:

For post-exposure years, significance of Wilcoxon signed-rank test p adjusted for multiple comparisons denoted by ** ($p<0.01$), * ($0.01 \leq p<0.05$) or - (NS)

ESM Table 8: Estimated fold-change (95 % CI) in HbA_{1c} yearly pre-exposure and for FM-exposed time compared to the counterfactual- overall and stratified by SIMD quintile from mixed models adjusting for sex, baseline HbA_{1c}, age and diabetes duration at FM initiation

Variable	Q1	Q2	Q3	Q4	Q5
Time effect (years)	0.99 (0.99; 1.00)	0.99 (0.99; 0.99)	0.99 (0.99; 0.99)	0.99 (0.99; 0.99)	0.99 (0.99; 0.99)
**FM usage (ref=no FM usage)**					
0-1 year	0.94 (0.94; 0.95)	0.95 (0.94; 0.95)	0.94 (0.94; 0.95)	0.94 (0.94; 0.95)	0.94 (0.93; 0.94)
1-2 years	0.99 (0.98; 1.01)	0.99 (0.98; 1.00)	0.99 (0.98; 1.00)	0.98 (0.97; 0.99)	0.97 (0.96; 0.98)
2 or more years	0.96 (0.92; 0.99)	0.99 (0.96; 1.02)	0.99 (0.96; 1.01)	1.00 (0.98; 1.02)	0.99 (0.98; 1.01)
Number of observations	9506	12795	12725	13843	14461
Number of individuals	1757	2330	2297	2455	2514

ESM Table 9: Absolute within-person differences in HbA_{1c} (mmol/mol), with respect to baseline, by time from FM initiation (years), stratified by prior pump usage - N and median (IQR).Data are dual reported in HbA_{1c} percentage units

Time from FM initiation (yrs)	No prior pump/cgm use	Prior pump use
**Pre-FM**		
4-5 yrs bef.	0.50(-5.50,7.50)|6707<br>0.0(-0.5,0.7)%	2.50(-4.00,9.00)|2374<br>0.2(-0.4,0.8)%
3-4 yrs bef.	0.50(-5.00,6.50)|6981<br>0.0(-0.5,0.6)%	1.00(-4.50,7.00)|2539<br>0.1(-0.4,0.6)%
2-3 yrs bef.	0.50(-4.00,5.50)|7258<br>0.0(-0.4,0.5)%	1.00(-3.50,5.50)|2640<br>0.1(-0.3,0.5)%
1-2 yrs bef.	ref.|7677	ref.|2737
0-1 yr bef.	ref.|9024	ref.|2810
**Post-FM**		
0-1 yr aft.	-3.00(-10.50,2.00)|8082**<br>-0.3(-1.0,0.2)%	-1.50(-6.00,3.00)|2679**<br>-0.1(-0.5,0.3)%
1-2 yrs aft.	-2.00(-9.00,4.50)|3628**<br>-0.2(-0.8,0.4)%	0.00(-5.00,5.50)|1672-<br>0.0(-0.5,0.5)%
2+ yrs aft.	-4.00(-11.00,3.00)|470**<br>-0.4(-1.0,0.3)%	-1.00(-6.50,4.00)|288**<br>-0.1(-0.6,0.4)%

ESM Table 10: Estimated fold-change (95 % CI) in HbA_{1c} yearly pre-exposure and for FM-exposed time compared to the counterfactual- stratified by prior pump usage from mixed models adjusting for sex, age, baseline HbA_{1c}, and diabetes duration at FM initiation

Variable	Prior pump use=No	Yes
Time effect (years)	0.99 (0.99; 0.99)	0.99 (0.99; 0.99)
**FM usage (ref=no FM usage)**		
0-1 year	0.93 (0.92; 0.93)	0.99 (0.98; 0.99)
1-2 years	0.97 (0.96; 0.97)	1.03 (1.03; 1.04)
2 or more years	0.96 (0.95; 0.98)	1.05 (1.03; 1.07)
Number of observations	46635	16695
Number of individuals	8684	2669

ESM Table 11: Absolute within-person differences in HbA_{1c} (mmol/mol), with respect to baseline, by time from FM initiation (years), stratified by prior completed diabetes education programme - N and median (IQR). Data are dual reported in HbA_{1c} percentage units

Time from FM initiation (yrs)	Prior education=No	Yes
**Pre-FM**		
4-5 yrs bef.	1.00(-6.00,7.50)|5948<br>0.1(-0.5,0.7)%	2.00(-4.50,8.50)|3133<br>0.2(-0.4,0.8)%
3-4 yrs bef.	0.50(-5.00,6.50)|6288<br>0.0(-0.5,0.6)%	1.00(-4.50,7.00)|3232<br>0.1(-0.4,0.6)%
2-3 yrs bef.	0.50(-4.00,5.50)|6612<br>0.0(-0.4,0.5)%	1.00(-3.50,5.50)|3286<br>0.1(-0.3,0.5)%
1-2 yrs bef.	ref.|7017	ref.|3397
0-1 yr bef.	ref.|8253	ref.|3581
**Post-FM**		
0-1 yr aft.	-2.50(-9.00,3.00)|7454**<br>-0.2(-0.8,0.3)%	-3.00(-9.00,1.50)|3307**<br>-0.3(-0.8,0.1)%
1-2 yrs aft.	-1.00(-7.62,5.00)|3680**<br>-0.1(-0.7,0.5)%	-1.50(-7.50,4.00)|1620**<br>-0.1(-0.7,0.4)%
2+ yrs aft.	-2.00(-9.00,3.50)|563**<br>-0.2(-0.8,0.3)%	-3.00(-11.12,3.50)|195**<br>-0.3(-1.0,0.3)%

ESM Table 12: Estimated fold-change (95 % CI) in HbA_{1c}yearly pre-exposure and for FM-exposed time compared to the counterfactual- stratified by prior completed diabetes education programme, from mixed models adjusting for sex, age, baseline HbA_{1c} and diabetes duration at FM initiation

Variable	Prior completed education=No	Yes
Time effect (years)	0.99 (0.99; 0.99)	0.99 (0.99; 0.99)
**FM usage (ref=no FM usage)**		
0-1 year	0.94 (0.93; 0.94)	0.95 (0.95; 0.96)
1-2 years	0.98 (0.97; 0.99)	0.99 (0.98; 1.00)
2 or more years	0.98 (0.97; 1.00)	1.01 (0.99; 1.03)
Number of observations	43391	19939
Number of individuals	8007	3346

ESM Table 13: Absolute within-person differences in HbA_{1c} (mmol/mol), with respect to baseline, by time from FM initiation (years), stratified by early adoption of FM - N and median (IQR). Data are dual reported in HbA_{1c} percentage units.

<table>
<tr><th>Time from
FM initiation (yrs)</th><th>Early adopter=No</th><th>Yes</th></tr>
<tr><td>Pre-FM</td><td></td><td></td></tr>
<tr><td>4-5 yrs bef.</td><td>1.00(-5.00,8.00)|8429
0.1(-0.5,0.7)%</td><td>2.00(-4.00,8.00)|652
0.2(-0.4,0.7)%</td></tr>
<tr><td>3-4 yrs bef.</td><td>1.00(-5.00,6.50)|8819
0.1(-0.5,0.6)%</td><td>1.00(-4.00,7.00)|701
0.1(-0.4,0.6)%</td></tr>
<tr><td>2-3 yrs bef.</td><td>0.50(-4.00,5.50)|9171
0.0(-0.4,0.5)%</td><td>1.00(-4.00,5.50)|727
0.1(-0.4,0.5)%</td></tr>
<tr><td>1-2 yrs bef.</td><td>ref.|9603</td><td>ref.|811</td></tr>
<tr><td>0-1 yr bef.</td><td>ref.|10930</td><td>ref.|904</td></tr>
<tr><td>Post-FM</td><td></td><td></td></tr>
<tr><td>0-1 yr aft.</td><td>-2.50(-9.00,2.50)|9874**
-0.2(-0.8,0.2)%</td><td>-3.00(-9.00,1.00)|887**
-0.3(-0.8,0.1)%</td></tr>
<tr><td>1-2 yrs aft.</td><td>-0.50(-7.00,5.00)|4508**
0.0(-0.6,0.5)%</td><td>-4.00(-10.50,2.00)|792**
-0.4(-1.0,0.2)%</td></tr>
<tr><td>2+ yrs aft.</td><td>1.00(-7.25,6.25)|119-
0.1(-0.7,0.6)%</td><td>-3.00(-9.44,3.00)|639**
-0.3(-0.9,0.3)%</td></tr>
</table>

ESM Table 14: Estimated fold-change (95 % CI) in HbA_{1c} yearly pre-exposure and for FM-exposed time compared to the counterfactual- stratified by early adopter status from mixed models adjusting for sex, age, baseline HbA_{1c}, and diabetes duration at FM initiation

Variable	Early adopter=No	Yes
Time effect (years)	0.99 (0.99; 0.99)	0.99 (0.98; 0.99)
**FM usage (ref=no FM usage)**		
0-1 year	0.94 (0.94; 0.95)	0.93 (0.92; 0.94)
1-2 years	0.99 (0.98; 1.00)	0.95 (0.93; 0.96)
2 or more years	1.01 (0.99; 1.04)	0.98 (0.96; 0.99)
Number of observations	57077	6253
Number of individuals	10441	912

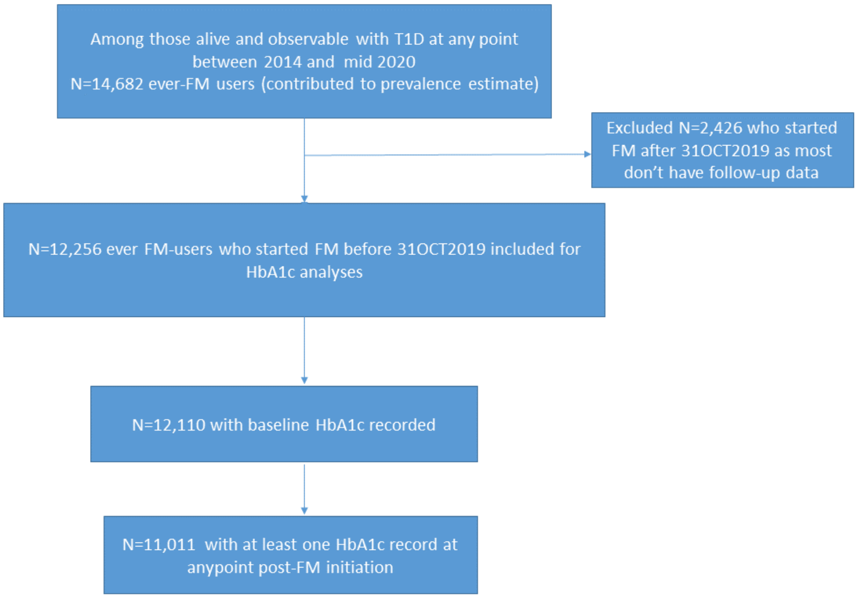

ESM Figure 1: Study sample-size flowchart

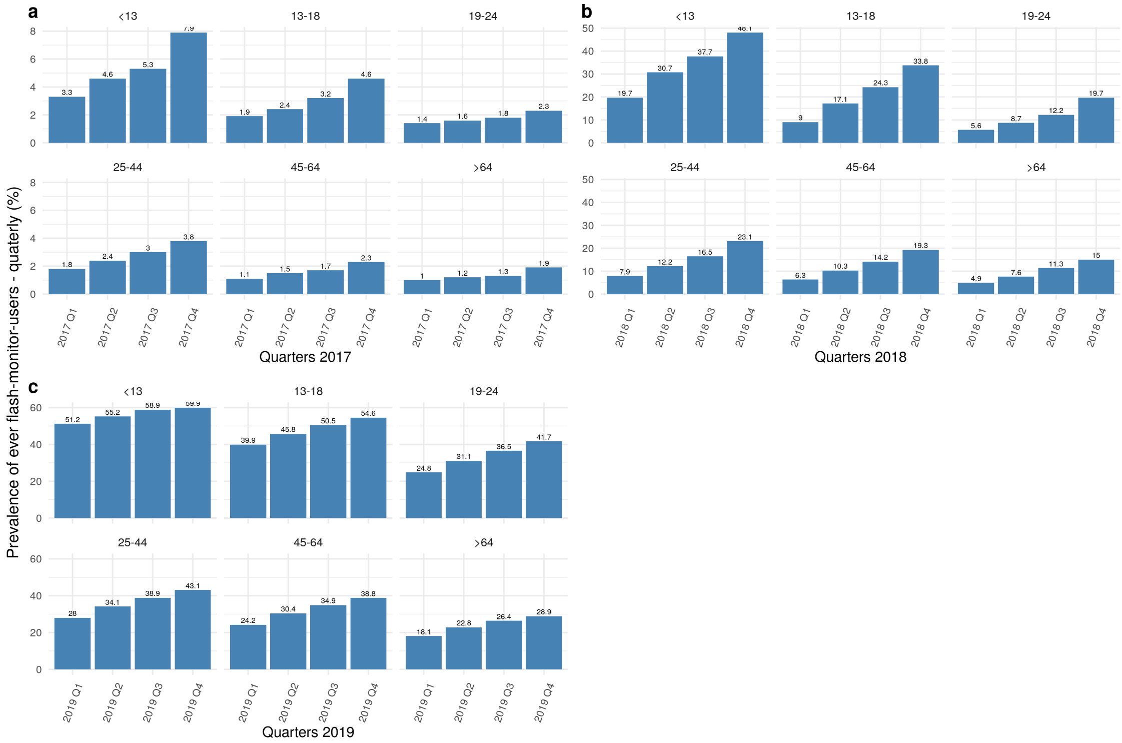

ESM Figure 2: Quaterly prevalence of ever FM users - 2017 (a), 2018 (b), 2019 (c)

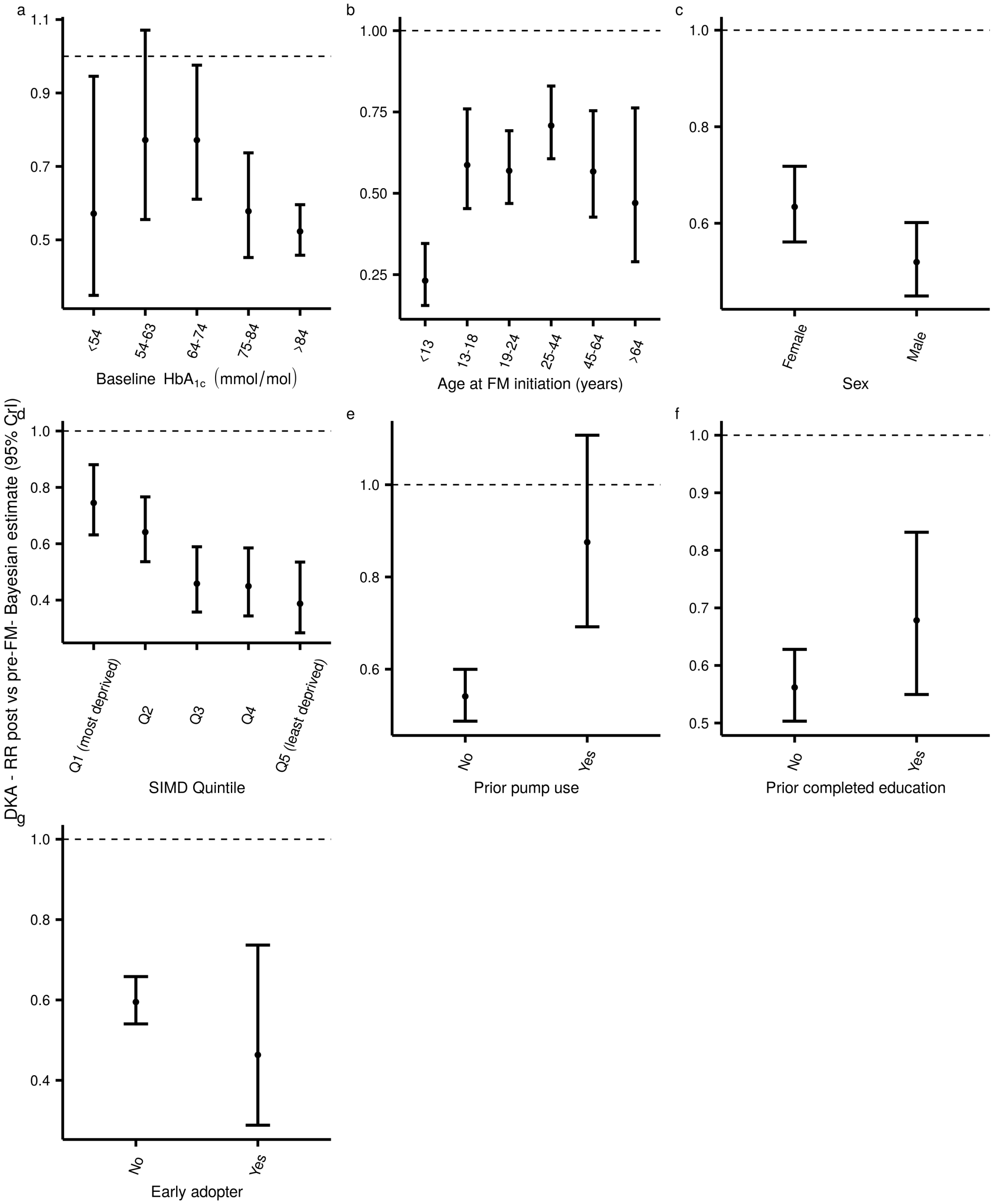

ESM Figure 3: DKA- Rate Ratios- post vs pre-FM initiation- Bayesian posterior mean (95%CrI), adjusted for pre-exposure trend, baseline HbA_{1c}, age, sex and diabetes duration - stratified by baseline HbA_{1c} (a), age band at FM initiation (b), sex (c), SIMD quintile (d), prior pump usage (e), prior diabetes education programme (f), early adoption (g)
